# Supplementary material for: Downregulation of miR-1388 Regulates the Expression of Antiviral Genes via Tumor Necrosis Factor Receptor (TNFR)-Associated Factor 3 Targeting Following poly(I:C) Stimulation in Silver Carp (Hypophthalmichthys molitrix)
Source: Biomolecules. 2024 Jun 14;14(6):694. doi: 10.3390/biom14060694 (PMC11201635; doi:10.3390/biom14060694)
Supplement: Supplementary file 1 [file biomolecules-14-00694-s001.zip › biomolecules-3021611-supplementary.pdf]

Table S1. Primer sequence information in this study.

Primers	Sequences (5′-3′)	Tm	product size	amplification efficiency
**plasmid construction**				
(GLO)TRAF3-3′UTR-SacI-F	CGAGCTCAGACGACACCATCTTCATCAAA	62.1	253	93%
(GLO)TRAF3-3′UTR-XholI-R	TGCCTCGAGCCTCCTCTTAAAGACCGTAACTAAA	64.6		
(GLO)TRAF3-3′UTR-F-MT	TTGTAGTGACTTGAGCCGTCAGCTTGTGCTCG	62.97	5674	90%
(GLO)TRAF3-3′UTR-R-MT	GGCTCAAGTCACTACAAATCCAAACCTGTCCTGGG	64.57		
(GFP)TRAF3-3′UTR-SacI-F	CGAGCTCAGACGACACCATCTTCATCAAA	62.1	274	95%
(GFP)TRAF3-3′UTR-SalI-R	ACGCGTCGACCCTCCTCTTAAAGACCGTAACTAAA	65.9		
(pcDNA)TRAF3-HindIII-F	TGACGACAAGAAGCTACTGATTCATGTCATGTCCGCAGGGC	69.6	1972	94%
(pcDNA)TRAF3-XholI-R	TAGATGCATGCTCGACTCCTTGCTCCGTAGATCCAG	67.8		
**RT-PCR**				
miR-1388-F	GCGAGGACTGTCCAACCTG	59.1	62	95%
miR-1388-R	AGTGCAGGGTCCGAGGTATT	58.5		
GAPDH-F	GGGATTGTCGCTCATCTATCTT	54	113	98%
GAPDH-R	GTTGCTGTACCCAAACTCATTG	54.2		
U6-F	GCTTCGGCAGCACATATACTAA	54.7	89	98%
U6-R	GCTTCACGAATTTGCGTGTCAT	56.4		
TRAF3-F	CACGGGCTATTTCGGCTATAA	53.1	103	95%
TRAF3-R	CGCATGACCACGAAGAAGA	55.6		
IFN-I-F	GAGGACCAGGTGAAGTTTCTT	53.1	121	91%
IFN-I-R	CAGTCTGTAGGTTCCACTGC	54.5		
MX1-F	ACGCCTCACAGACTATGTGCC	60.1	105	92%
MX1-R	TCAGCAGTTTGACCACATCTGCC	60.2		
PKR-F	ATCTGAGGATGCACGACTCTCATC	58.5	96	91%
PKR-R	GCTCGCACAAGGACTTATGGC	59.4		
Viperin-F	GACCACTCCAAGCAGTGTAAA	54	107	93%
Viperin-R	TCAATAGGCAAGACGAACGAG	55.1		

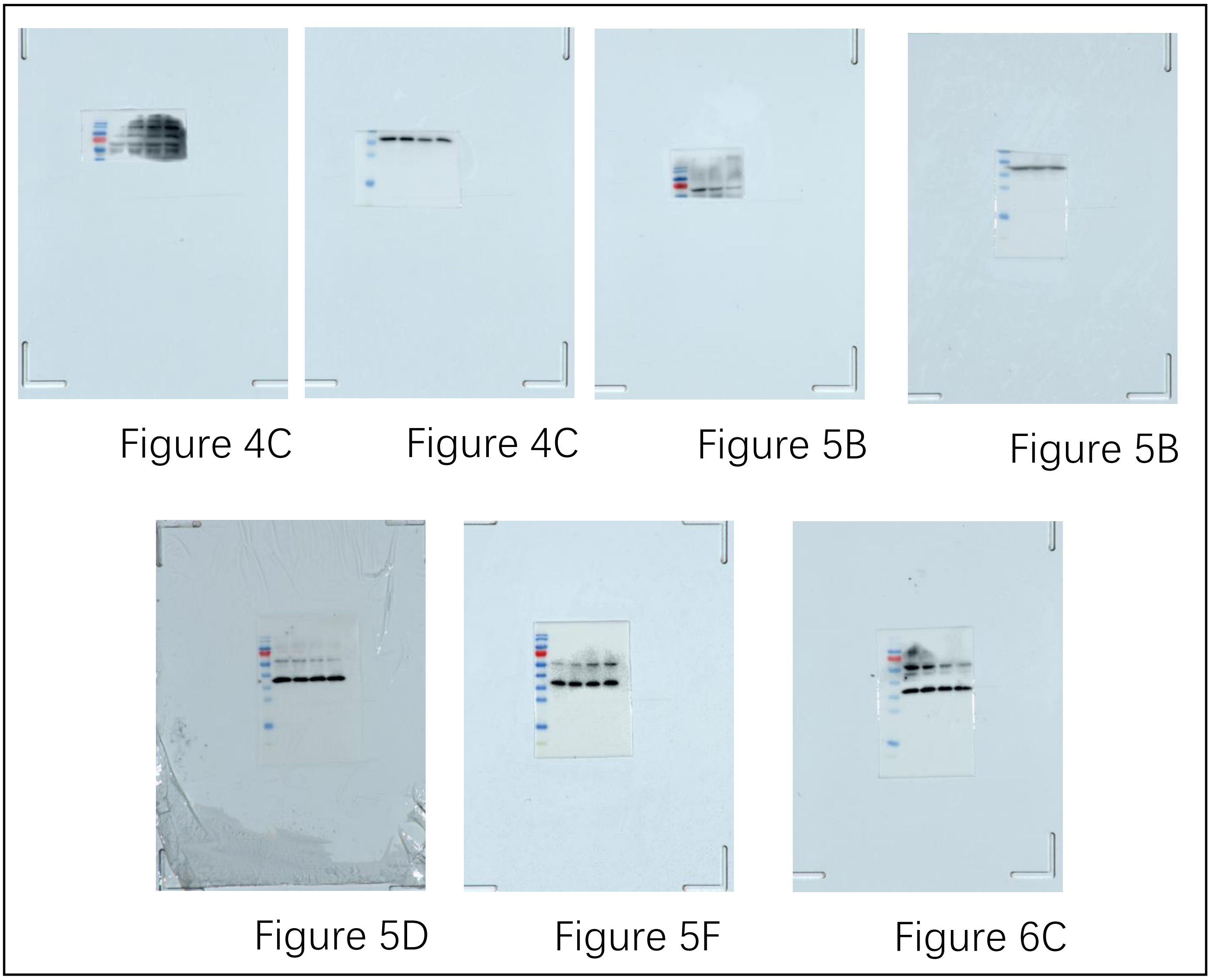

Figure S1. Original Images of Western Blots.
